# Metagenomics reveals the habitat specificity of biosynthetic potential of secondary metabolites in global food fermentations

**DOI:** 10.1186/s40168-023-01536-8

**Published:** 2023-05-20

**Authors:** Rubing Du, Wu Xiong, Lei Xu, Yan Xu, Qun Wu

**Affiliations:** 1 Lab of Brewing Microbiology and Applied Enzymology, The Key Laboratory of Industrial Biotechnology, Ministry of Education, State Key Laboratory of Food Science and Technology, School of Biotechnology, Jiangnan University, Wuxi, 214122 Jiangsu People’s Republic of China; 2 Laboratory of Bio-Interactions and Crop Health, Jiangsu Provincial Key Lab of Solid Organic Waste Utilization, Jiangsu Collaborative Innovation Center of Solid Organic Wastes, Educational Ministry Engineering Center of Resource-Saving Fertilizers, Nanjing Agricultural University, Nanjing, 210095 Jiangsu People’s Republic of China

**Keywords:** Food fermentation, Biosynthetic gene clusters, Metagenome-assembled genomes, Human health, Metagenomic sequencing, Biological activity

## Abstract

**Background:**

Fermented foods are considered to be beneficial for human health. Secondary metabolites determined by biosynthetic gene clusters (BGCs) are precious bioactive compounds with various biological activities. However, the diversity and distribution of the biosynthetic potential of secondary metabolites in global food fermentations remain largely unknown. In this study, we performed a large-scale and comprehensive investigation for the BGCs in global food fermentations by metagenomics analysis.

**Results:**

We recovered 653 bacterial metagenome-assembled genomes (MAGs) from 367 metagenomic sequencing datasets covering 15 general food fermentation types worldwide. In total, 2334 secondary metabolite BGCs, including 1003 novel BGCs, were identified in these MAGs. Bacillaceae, Streptococcaceae, Streptomycetaceae, Brevibacteriaceae and Lactobacillaceae contained high abundances of novel BGCs (≥ 60 novel BGCs). Among 2334 BGCs, 1655 were habitat-specific, originating from habitat-specific species (80.54%) and habitat-specific genotypes within multi-habitat species (19.46%) in different food fermentation types. Biological activity analysis suggested that 183 BGC-producing secondary metabolites exhibited high probabilities of antibacterial activity (> 80%). These 183 BGCs were distributed across all 15 food fermentation types, and cheese fermentation contained the most BGC number.

**Conclusions:**

This study demonstrates that food fermentation systems are an untapped reservoir of BGCs and bioactive secondary metabolites, and it provides novel insights into the potential human health benefits of fermented foods.

Video Abstract

**Supplementary Information:**

The online version contains supplementary material available at 10.1186/s40168-023-01536-8

## Background

Fermented foods, important part of the human diet, have been produced and consumed since the development of human civilizations [1]. There are more than 200 fermented foods worldwide, for example cheese, kefir, kimchi, bean paste and soy sauce [1]. The consumption of fermented foods is increasing [2], and it has been recommended that fermented foods should be included in national dietary guidelines/recommendations because of their health benefits [3]. Fermented foods are closely associated with human health via contributing not only the essential nutrients but also the bioactive metabolites produced by microorganisms in fermented foods [4, 5]. Many fermented foods are produced in stressful environments, such as high salinity [6, 7], high temperature [8], high acidity [9] and high ethanol content [10]. These unique environments result in a variety of specific microorganisms [11], which are the main producers of these bioactive metabolites [4]. Thus, revealing the microorganisms and biosynthetic potential of bioactive metabolites would be important to elucidate the health benefits of fermented foods.

Secondary metabolites include ribosomally synthesised and post-translationally modified peptide (RiPP), nonribosomal peptide, polyketide and terpene [12]. Although not required for normal cell growth, they have multiple physiological functions, including nutrient acquisition, communication and inhibition, allowing their producers to thrive in specific habitats [13, 14]. They also have various bioactivities, such as antibacterial, antiviral and anti-inflammatory activities [15, 16]. Therefore, it would be beneficial to unveil the biosynthetic potential of secondary metabolites in food fermentations.

The biosynthetic potential of secondary metabolites can be revealed by mining biosynthetic gene clusters (BGCs) related to secondary metabolites [17]. In silico genome analysis facilitates large-scale mining of BGCs [18], and a lot of BGCs have been identified in some microbial ecosystems, such as the human gut [19], ocean [20] and soil [13] ecosystems. Recently, 210 bacteriocin-producing gene clusters were assigned in cheese fermentation [21], and 55 putative bacteriocin-producing gene clusters were assigned in different fermented food samples [22], indicating the biosynthetic potential of secondary metabolites in food fermentations. However, the extent and distribution of the biosynthetic potential of secondary metabolites in global food fermentations are unclear.

In this study, we collected metagenomic sequencing data from 367 samples involving 15 food fermentation types from 4 continents. We performed metagenomic binning analysis to recover metagenome-assembled genomes (MAGs) from these samples and comprehensively characterise the distribution of BGCs in different food fermentation types. We also assessed the novelty and uniqueness of BGCs by comparing them with those in the BiG-FAM database and with those in the human gut, ocean and soil ecosystems. These findings greatly improve our understanding of the biosynthetic potential of secondary metabolites in global food fermentations and facilitate elucidating the health benefits of fermented foods.

## Methods

### Metagenomic data collection

The metagenomic sequencing data were collected by searching the keyword ‘food’ in the NCBI SRA database in July 2020. Meanwhile, we also searched studies using keywords such as ‘food’, ‘cheese’, ‘kefir’, ‘Chinese liquor’, ‘nunu’, ‘kombucha’, ‘koumiss’, ‘wine’, paste’, ‘cocoa’, ‘yoghurt’, ‘kimchi’, ‘sauce’, ‘fermented meat’ and ‘sourdough’ in Web of Science and Google Scholar in July 2020. The metagenomic sequencing data mentioned in these papers were collected. A total of 367 metagenomic sequencing data were obtained. There were 2 library layouts (pair-end sequencing, *n* = 314; single-end sequencing, *n* = 53) and 10 sequencing platforms (454 GS FLX Titanium, *n* = 12; BGISEQ-500, *n* = 10; HiSeq X Ten, *n* = 20; Illumina HiSeq 1500, *n* = 10; Illumina HiSeq 2000, *n* = 7; Illumina HiSeq 2500, *n* = 11; Illumina HiSeq 4000, *n* = 66; Illumina MiSeq, *n* = 64; Ion Torrent Proton, *n* = 6; NextSeq 500, *n* = 161). All metagenomic sequencing data were obtained using SRA-tools fastq-dump (https://github.com/ncbi/sra-tools). The detailed information of each sample was described in Supplementary Data 1.

### Metagenomic sequencing data assembly and binning

#### Assembly

Raw reads of each metagenomic sequencing data were filtered to remove adapter sequences and low-quality reads (quality score < 20) using Trim Galore (v. 0.5.0) (http://www.bioinformatics.babraham.ac.uk/projects/trim_galore/) with default parameters. The read quality was checked using FastQC (v. 0.11.8) (https://www.bioinformatics.babraham.ac.uk/projects/fastqc/). A total of 1.4 Tb of clean reads were retained. The different assemblers (MEGAHIT and metaSPAdes) and different processes (single-sample and co-assembly) could affect the quantity and quality of MAGs [23] in subsequent binning analysis. Therefore, the clean reads of each sample were used for assembly using MEGAHIT (v. 1.1.3) [24] and metaSPAdes (v. 3.13.0) [25] with default parameters. Then, the clean reads from the same study were mixed into one fastq file (for pair-end sequencing data, upstream and downstream sequencing data files were mixed separately). The mixed clean reads were co-assembled using MEGAHIT (v. 1.1.3) [24].

#### Metagenomic binning

The contigs from both co-assembly and single-sample assembly were filtered based on the sequence length. The contigs with sequence length > 1500 bp were retained by seqtk (https://github.com/lh3/seqtk) and used for metagenomic binning. For metagenomic binning analysis, the clean reads were mapped to corresponding contigs using Bowtie2 (v. 2.4.4) [26]. Samtools (v. 1.7.0) was used to convert mapped results into BAM format [27]. Then, the BAM files were sorted and indexed using SAMtools (v. 1.7.0) [27]. The resulting sorted BAM files were used for metagenomic binning based on the sequence characteristics and coverage depth using MaxBin2 (v. 2.2.7) [28], MetaBAT2 (v. 2:2.15) [29] and CONCOCT (v. 1.1.0) [30]. DAS Tool (v. 1.1.2) [31] was then applied to integrate MAGs generated from different methods.

The completeness and contamination of all MAGs were estimated using CheckM (v. 1.0.12) [32] based on the lineage_wf workflow. The MAGs with medium and high qualities (completeness ≥ 50% and contamination ≤ 10%) were retained. The retained MAGs were classified into 15 datasets based on food fermentation types. Then, MAGs from each food fermentation type were dereplicated using fastANI algorithm in dRep (v. 3.2.2) [33] at the threshold of 99% average nucleotide identity (ANI) (strains level) with at least 25% overlap between genomes. Meanwhile, to enhance the diversity of the dataset, the publicly available 328 MAGs in cheese fermentation [21] and 29 MAGs in cocoa fermentation [34] were compared with the MAGs in cheese and cocoa fermentations in this study, respectively. We removed the repeated MAGs between publicly available and our MAGs based on 99% ANI using dRep (v. 3.2.2) [33]. There were 27 and 18 different bacterial MAGs in cheese fermentation [21] and cocoa fermentation [34], respectively. These different MAGs were added in the corresponding food fermentation types in this study. A total of 653 bacterial MAGs were obtained from 15 food fermentation types. These 653 MAGs were nonredundant MAGs recovered after combination with public data and dereplication. All MAGs were taxonomically annotated using GTDB-Tk (v. 0.1.6) [35] based on the Genome Taxonomy Database (http://gtdb.ecogenomic.org/), and the standardised taxonomic labels were obtained. The detailed commands in metagenomic assembly and binning analysis are available at https://github.com/durubing-jn/food-fermentation-mategenome.

### Phylogenetic analysis

The phylogenetic tree was built based on the sequences of 653 MAGs. The aligned protein sequences were produced using GTDB-Tk (v. 0.1.6) [35] and edited using BMGE (v. 1.12) [36] to select phylogenetically informative regions. FastTree 2 (v. 2.1.10) [37] was used to infer phylogenetic trees based on the default parameters. The phylogenetic tree was edited and visualised using the interactive Tree Of Life (iTOL) (v. 5) [38].

### Analysis of habitat-specific species and habitat-specific genotypes

A total of 653 nonredundant bacterial MAGs, which were defined based on 99% ANI, were used to analyse habitat-specific species. Species were classified based on species-level thresholds (95% ANI) using dRep (v. 3.2.2) [33]. Species present in only one food fermentation type were considered as habitat-specific species. MAGs present in only one food fermentation type were considered as habitat-specific genotypes.

### Biosynthetic gene cluster analysis

The BGCs in the MAGs were identified using antiSMASH (v. 6.0) [39]. Parameters were as follows: –taxon bacteria, –genefinding-tool prodigal, –cb-knownclusters, –cc-mibig and –fullhmmer.

### Distribution of biosynthetic gene clusters in food fermentations

Clustering analysis was performed using BiG-SCAPE (v. 1.1.0) with the PFAM database (v. 31.0) [40]. The gbk files of BGCs, which were outputted by antiSMASH, were used as input. Analysis was performed using default settings with ‘ − mibig’. The BGCs from antiSMASH analysis, as well as 1923 previously characterised BGCs from the MiBiG database (v. 2.0), were subjected to clustering analysis. The BGCs were clustered into gene cluster families (GCFs) based on the similarity network of BGC sequences with a default score cutoff (*c* = 0.3). The GCFs consisting of BGCs from the antiSMASH analysis and MiBiG database were considered as known GCFs. The type of information of each BGC and GCF was obtained from the results file that was outputted by BiG-SCAPE. The GCFs consisting of BGCs from the same food fermentation were defined as habitat-specific, and the corresponding BGCs were habitat-specific. The GCFs consisting of BGCs from different food fermentations were defined as multi-habitat, and the corresponding BGCs were multi-habitat.

### Assessment of novelty for biosynthetic gene clusters in food fermentations

To calculate the novelty of BGCs in food fermentations, BiG-SLiCE (v. 1.1.0) [41] was run in query mode with the BiG-FAM database and *t* = 900 as the threshold. The resulting BiG-SLiCE distance indicated how closely a given BGC was related to previously computed GCFs, with a greater BiG-SLiCE distance indicating greater novelty. For this analysis, we highlighted values of BiG-SLiCE distance > *t* because it was previously suggested as the cutoff for novel BGCs [41].

### Comparison of biosynthetic gene clusters between food fermentations and other ecosystems

The BGCs from the human gut, ocean and soil ecosystems were collected from available resources from published studies [13, 19, 20]. In total, 8901 BGCs from the human gut ecosystem were downloaded from the HRGM web server (https://www.mbiomenet.org/HRGM/) [19], 39,055 BGCs from the ocean ecosystem were obtained from the Ocean Microbiomics Database (https://microbiomics.io/ocean/) [20], and 7529 BGCs from the soil ecosystem were obtained from 1334 geneome stored in 10.6084/m9.Fig.share.10045988[13]. All BGCs were saved in gbk format.

To calculate the difference in BGCs between food fermentations and other ecosystems, we performed a BiG-SLiCE clustering analysis using the human gut, ocean and soil ecosystem BGCs as input. Then, we performed a query of BGCs from food fermentations against three preprocessed sets (the outputs of BiG-SLiCE clustering analysis), respectively, using *t* = 900 as the threshold. The BiG-SLiCE distance matrix of BGCs from food fermentations to the closest GCF from the three ecosystems was used for PCA analysis. PCA analysis was performed using SPSS Statistics 26 (IBM, Armonk, NY, USA).

### Network of biosynthetic gene clusters

To construct a network of BGCs, a sequence similarity matrix of BGCs was obtained from the BiG-SCAPE analysis with a default similarity score cutoff (*c* = 0.3). The network of BGCs was visualised and edited using Cytoscape (v. 3.8.2).

### Biological activity prediction of the product of biosynthetic gene clusters

The nucleotide sequence of each BGC was extracted from the output file of antiSMASH. The antibiotic resistance gene in a BGC was identified using Resistance Gene Identifier (v. 5.1.1) with the extracted nucleotide sequence as input [42]. The Comprehensive Antibiotic Resistance Database (v. 3.2.3) was used as reference data. The command line tool (cluster_function_prediction.py) was run using default parameters. The output files from antiSMASH (gbk format) and Resistance Gene Identifier (txt format) were used to predict biological activities of corresponding secondary metabolites by a developed machine learning model as described previously [43].

### Statistical analysis

The associations between phylogenetic distribution of MAGs and food fermentation groups were analysed using chi-squared test. The statistical difference for the number of BCGs per MAG between different habitats was analysed based on one-way ANOVA and Tukey HSD post hoc test. Wilcoxon rank-sum test was used to analyse BiG-SLiCE distances of BGCs from food fermentations compared with those from different ecosystems. *P*-value was used to evaluate statistical significance. One-way ANOVA and Tukey HSD post hoc test were performed using SPSS Statistics 26 (IBM, Armonk, NY, USA). Wilcoxon rank-sum test was performed using wilcox.test() function in R (v. 3.6.1). Chi-squared test was done using chisq.test() function in R (v. 3.6.1).

## Results

### Habitat specificity of microorganisms in food fermentations

**Fig. 1:**
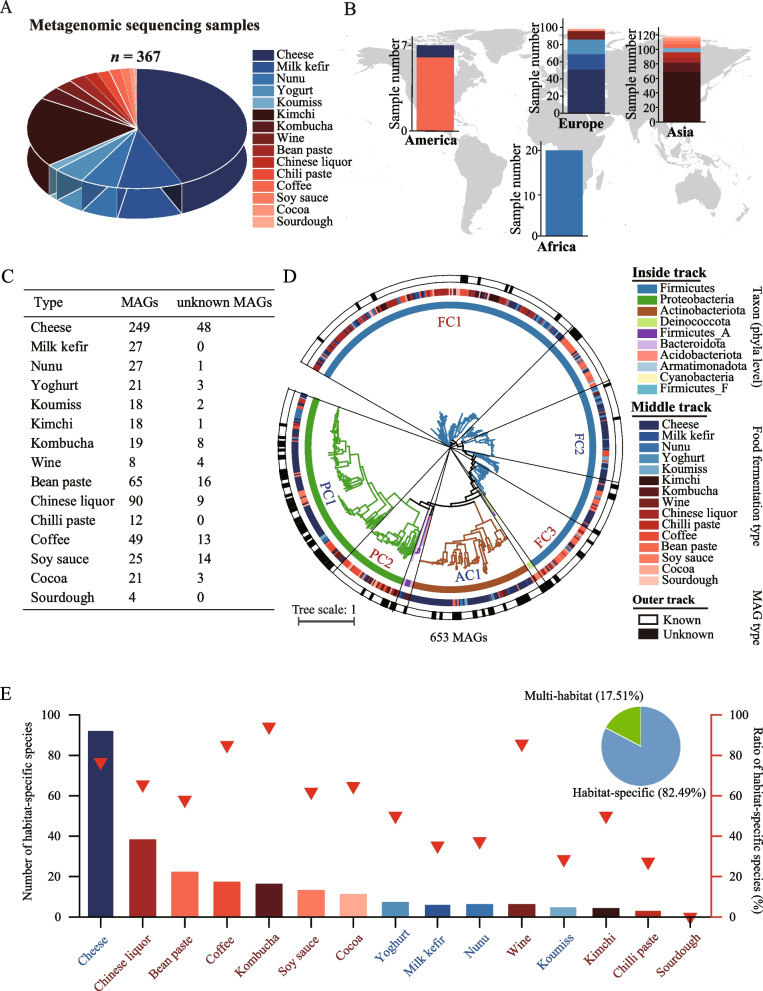
Distribution of metagenome-assembled genomes (MAGs) among different food fermentation types from 4 continents. **A** The abundance of food fermentation samples in different food fermentation types. **B** The number of food fermentation samples with known origins in four continents. **C** The number of all MAGs and unknown MAGs in different food fermentation types. MAGs, which could not be annotated by GTDB-tk (*ANI* < 95%), were defined as unknown MAGs. **D** Taxonomic annotation (assigned to species level) and phylogenetic tree of 653 bacterial MAGs. The clusters were classified based on the taxonomic classification of each MAG. FC1, Lactobacillaceae; FC2, Streptococcaceae; FC3, Bacillaceae, Bacillaceae_C, Bacillaceae_D, Bacillaceae_G, Anoxybacillaceae, Amphibacillaceae and Thermoactinomycetaceae; PC1, Gammaproteobacteria; PC2, Alphaproteobacteria; and AC1, Actinobacteriota. **E** Distribution of the habitat-specific species in different food fermentation types. The bars represent the numbers of habitat-specific species in each food fermentation type. The triangles represent the ratios of habitat-specific species number to the total species number in different food fermentation types. The pie chart represents the ratio of habitat-specific species in all food fermentation samples

We collected metagenomic sequencing data from 367 samples involving 15 food fermentation types from 4 continents (Supplementary Data 1). These samples included a milk-based fermentation group (cheese, milk kefir, nunu, yoghurt and koumiss) and a plant-based fermentation group (kimchi, kombucha, wine, Chinese liquor, chilli paste, coffee, soy sauce, bean paste, cocoa and sourdough) (Fig. 1A). The origin of 243 samples was obtained from the National Center for Biotechnology Information (NCBI) database (Fig. 1B). Ninety-eight samples were from Europe, of which 87.76% belonged to the milk-based fermentation group, and 118 samples were from Asia, of which 94.92% belonged to the plant-based fermentation group (Fig. 1B). These results revealed the geographical difference in food fermentation groups between Europe and Asia.

In total, 1.43 Tb of raw data was obtained from all food fermentation samples with an average sequencing depth of 9.59 log_10_ base/sample (Supplementary Fig. 1). A total of 17,524 MAGs were recovered from metagenomic sequencing data by metagenomic binning analysis. A total of 5557 MAGs met or exceeded medium quality (≥ 50% completeness and ≤ 10% contamination) according to MIMAG standards for MAGs [44] (Supplementary Fig. 2), and they were dereplicated into 608 nonredundant MAGs at 99% ANI. Compared with these 608 MAGs using 99% ANI, there were 27 and 18 different bacterial MAGs in publicly available 328 MAGs from cheese fermentation [21] and 29 MAGs from cocoa fermentation [34], respectively. These different bacterial MAGs were then added to the MAG dataset in the corresponding food fermentation type to enhance the diversity of the dataset in this study. A total of 653 nonredundant bacterial MAGs were finally obtained (Supplementary Data 2). These MAGs were assigned to 10 bacterial phyla (Supplementary Data 2). Most MAGs belonged to Firmicutes (382 MAGs, 58.50%), followed by Proteobacteria (169 MAGs, 25.88%) and Actinobacteriota (89 MAGs, 13.63%). In addition, 122 MAGs did not match any reference genomes (*ANI* < 95%) and were identified as unknown genomes at the species level (unknown MAGs), of which 4 MAGs could not be classified as known genera, and they were defined as novel genera. These 122 unknown MAGs came from 12 types of food fermentations (Fig. 1C), indicating the universality and richness of new species in food fermentations.

The distribution profile of 653 MAGs in food fermentation groups was analysed. We performed the association analysis between phylogenetic distribution of MAGs and food fermentation groups (Supplementary Data 3). At the phylum level, all Firmicutes_A, Firmicutes_F, Cyanobacteria, Acidobacteriota and Armatimonadota MAGs were specific to the plant-based fermentation group (Fig. 1D). Most Actinobacteriota MAGs (86.52%; *P* < 0.001) were from the milk-based fermentation group. Firmicutes and Proteobacteria MAGs were present in both fermentation groups. These two phyla were divided into five clusters [Firmicutes cluster (FC), FC1–FC3; Proteobacteria cluster (PC), PC1-PC2)] based on taxonomic classification. MAGs in FC1 (Lactobacillaceae) (62.94% in this cluster; *P* < 0.001); FC3 (Bacillaceae, Bacillaceae_C, Bacillaceae_D, Bacillaceae_G, Anoxybacillaceae, Amphibacillaceae, Thermoactinomycetaceae) (82.05%); and PC2 (Alphaproteobacteria) (93.18%; *P* < 0.001) were mainly from the plant-based fermentation group; MAGs in FC2 (Streptococcaceae) (86.15%; *P* < 0.001) and PC1 (Gammaproteobacteria) (71.2%; *P* < 0.001) were mainly from the milk-based fermentation group (Fig. 1D). These results revealed the phylogenetic distribution of MAGs between food fermentation groups.

To further analyse the distribution profile of MAGs at the species level among food fermentation types, we clustered MAGs using species-level thresholds (95% ANI), and all MAGs were assigned to 297 bacterial species. In total, 52 species (17.51%), present in more than one type of food fermentation, were multi-habitat species. Moreover, 245 species (82.49%), present in only one type of fermentation, were habitat-specific species. Cheese fermentation contained the most habitat-specific species (92 species), followed by Chinese liquor (38 species) and bean paste (22 species) fermentations (Fig. 1E). The ratio of habitat-specific species in kombucha fermentation was 94.12%, indicating that a large proportion of species in this food fermentation were different from those in other food fermentation types. These results revealed the habitat specificity of MAGs in food fermentation types.

### Habitat specificity of biosynthetic gene clusters in food fermentations

**Fig. 2:**
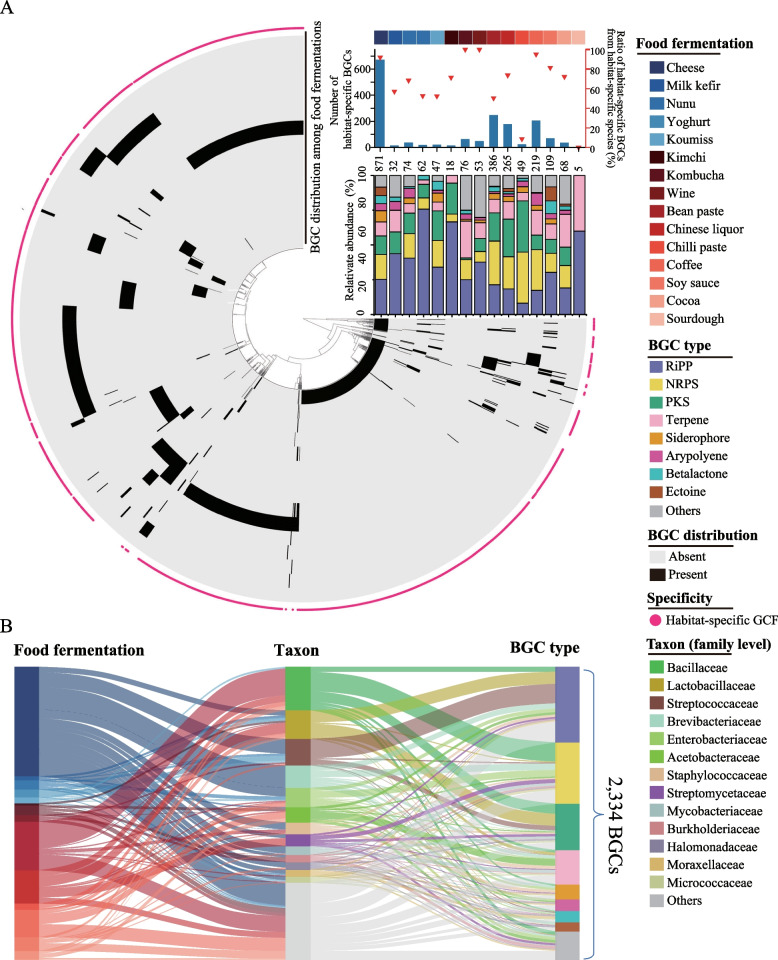
Distribution of biosynthetic gene clusters (BGCs) in different food fermentation types. **A** BGC overview among 15 food fermentation types. The central tree of the interface represents a hierarchical clustering dendrogram based on BGCs in a gene cluster family (GCF) among 15 food fermentation types. In the circle heat map, each layer represents the distribution of BGCs in different food fermentation types. The barplots represent the numbers of habitat-specific BGCs in different food fermentation types. The triangles represent the ratios of habitat-specific BGCs from habitat-specific species. The stacked columns represent the relative abundances of BGCs from different BGC types in different food fermentation types. The numbers above the stacked columns represent BGC amounts in different food fermentation types. **B** Sankey diagram showing the taxonomic origin (family level) of BGCs in different food fermentations and the composition of BGC type in different families. Species that are not included in the 13 BGC-rich families are combined and shown in Others. BGCs that are not included in the 8 dominant BGC types are combined and shown in Others

To determine the biosynthetic potential of secondary metabolites in food fermentations, we annotated BGCs of secondary metabolites within 653 bacterial MAGs. In total, 2334 BGCs were detected in 84.69% of MAGs (Supplementary Data 4). The number of BGCs ranged from 1 to 62 in different MAGs (Fig. 2A). Although the BGC numbers were discrepant among food fermentation types, only the number of BGC per MAG in bean paste fermentation was statistically different with that in cheese (*P* = 0.028), Chinese liquor (*P* = 0.015), kimchi (*P* = 0.013) and milk kefir (*P* = 0.002) fermentations (Supplementary Fig. 3). The highest BGC number in cheese fermentation was resulted from the largest number of available metagenomic data in cheese fermentation.

These BGCs were classified into 8 dominant types (BGC number per type > 50). RiPP, nonribosomal peptide synthetase (NRPS), polyketide synthase (PKS) and terpene were the four most dominant types of BGCs, containing 602, 488, 369 and 273 BGCs, respectively. Among all BGCs, 478 (20.48%) were identified from unknown MAGs, indicating the strong biosynthetic potential of secondary metabolites in unknown MAGs (Supplementary Fig. 4).

To reveal whether the distribution of BGCs was related to food fermentation type, we analysed the distribution of BGCs in 15 food fermentation types using clustering analysis based on the similarity of BGC sequences. All 2334 BGCs were clustered into 1415 GCFs using BiG-SCAPE (Supplementary Data 4). Among all BGCs, 1655 (70.91%) were present in only one type of food fermentation and were identified as habitat-specific BGCs. These habitat-specific BGCs were distributed in different food fermentation types with the number ranging from 3 to 672 (Fig. 2A). Cheese, bean paste and coffee fermentations each contained more than 200 habitat-specific BGCs, together accounting for 68.04% of the total habitat-specific BGCs.

We further analysed the taxonomic origin of BGCs. All BGCs were from 56 families. Among them, 13 families were identified as BGC-rich families (≥ 50 BGCs), and together, they contained 73.69% of the BGCs (Fig. 2B). Bacillaceae and Lactobacillaceae contained the most number of BGCs, with 346 and 227 BGCs, respectively. In addition, BGC composition differed in different families. Lactobacillaceae mainly contained RiPP and PKS (together 85.02% in this family), and Bacillaceae mainly contained NRPS and PKS (together 67.92%). Bacillaceae contained an average of 20 BGCs per MAG, and *Bacillus* *velezensis* MAG 282 contained the highest number of BGCs (45 BGCs). Although there were only 17 MAGs from Bacillaceae in these food fermentations, this family contributed the most amount of BGCs due to the strong contribution of BGCs in each MAG. Lactobacillaceae contributed the second most BGCs in food fermentations. Lactobacillaceae was the family with the highest number of species (73 species) and MAGs (197 MAGs) in these food fermentations, and 69.04% of MAGs in the Lactobacillaceae family contained BGCs (1.67 BGCs per MAG) (Supplementary Fig. 5). It suggested that although Lactobacillaceae MAG contained a low number of BGCs, Lactobacillaceae contributed a large amount of BGCs due to the largest number of MAGs in this family from these food fermentations.

Among the 1655 habitat-specific BGCs, 1333 BGCs (80.54%) originated from habitat-specific species (Supplementary Figs. 6 and 7). For example, the habitat-specific species *Brevibacterium aurantiacum* contributed 111 habitat-specific BGCs in cheese fermentation. The habitat-specific species *Bacillus glycinifermentans* contributed 13 habitat-specific BGCs in bean paste fermentation. In addition, 322 BGCs (19.46%) originated from habitat-specific genotypes within multi-habitat species (Supplementary Fig. 7). For example, *Lactococcus lactis* MAG 381 contributed 6 BGCs specifically in cheese fermentation, and *Bacillus velezensis* MAG 615 contributed 30 BGCs specifically in bean paste fermentation. These results indicated that the habitat specificity of BGCs might be driven by both habitat-specific species and habitat-specific genotypes within multi-habitat species in different food fermentation types. The effects of these driving factors were different in different food fermentation types. For instance, the habitat-specific BGCs in kombucha and wine fermentations all originated from habitat-specific species, and a large proportion of habitat-specific BGCs originated from habitat-specific species (> 70%) in cheese, kimchi, Chinese liquor, coffee, soy sauce and cocoa fermentations. However, the proportions of habitat-specific BGCs from habitat-specific species only reached 68.42%, 57.14%, 52.63%, 52.38%, 50.40% and 8.70% in nunu, milk kefir, yoghurt, koumiss, bean paste and chilli paste fermentations, respectively (Fig. 2A). This differentiation can be related to the divergent compositions of microbiota in different food fermentation types.

### The novelty of biosynthetic gene clusters in food fermentations

**Fig. 3:**
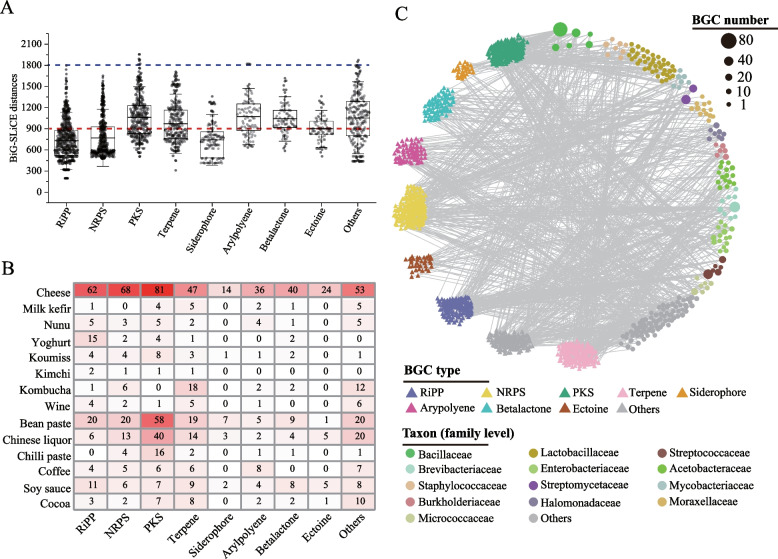
Novel biosynthetic gene clusters (BGCs) and their distributions in food fermentations. **A** BiG-SLiCE distance of BGCs in different BGC types compared with BGCs from FAM database. The red and blue dotted lines represent the BiG-SLiCE distances of 900 and 1800, respectively. The black lines in the boxplots are the average BiG-SLiCE distances of different BGC types. **B** The amount of novel BGCs from different BGC types in different food fermentation types. The numbers in the heat map represent the novel BGC counts. **C** Taxonomic origin of novel BGCs. The triangles and circles represent BGCs and bacterial species, respectively. The size of the circle represents the BGC number in the respective species. BGCs that are not included in the 8 dominant BGC types are combined and shown in Others. Species that are not included in the 13 BGC-rich families are combined and shown in Others

To evaluate the novelty of BGCs in food fermentations, we compared BGCs in food fermentations with those in the BiG-FAM database comprising 1.2 million known BGCs [45]. We calculated their BiG-SLiCE distances using BiG-SLiCE’s query mode. Notably, 1003 of 2334 BGCs (42.97%) in food fermentations had BiG-SLiCE distances of ≥ 900, indicating that they were distantly related to this reference dataset and were novel BGCs. Twelve BGCs had BiG-SLiCE distances of ≥ 1800, indicating that they were extremely divergent BGCs (Fig. 3A).

We analysed the distribution of these 1003 novel BGCs in food fermentation types (Fig. 3B). These novel BGCs were present in 14 types of food fermentation with the ratio of novel BGCs ranging from 16.44 to 56.25% (Supplementary Fig. 8), indicating a widespread distribution of novel BGCs in food fermentations. Cheese fermentation contained the most novel BGCs (425 BGCs) (Supplementary Fig. 8). These results suggested that the constitution of novel BGCs was divergent in different food fermentation types. However, there was no statistical difference for the number of novel BGC per MAG between different food fermentation types (*P* > 0.05). The highest novel BGC number in cheese fermentation might be resulted from the largest number of available metagenomic data in cheese fermentation.

The taxonomic origin of these 1003 novel BGCs was analysed (Fig. 3C). These novel BGCs were from 236 species, of which 19.24% were unknown species. This ratio was consistent with the ratio (20.61%) of unknown species to species associated with all BGCs, indicating that novel BGCs might not be specifically contributed by unknown species in food fermentations (Supplementary Fig. 4). These novel BGCs were present in all 13 BGC-rich families. Besides 3 well-known BGC-rich families (Bacillaceae, Streptococcaceae and Streptomycetaceae) that had high abundances of novel BGCs (≥ 60 novel BGCs), Brevibacteriaceae and Lactobacillaceae also had high abundances of novel BGCs (75 and 72, respectively). The proportions of novel BGCs in Bacillaceae, Streptococcaceae, Brevibacteriaceae, Lactobacillaceae and Streptomycetaceae reached 46.24%, 38.21%, 41.90%, 31.72% and 65.96%, respectively (Supplementary Fig. 9). In Brevibacteriaceae, all species contained novel BGCs, and *B.* *aurantiacum* contained the most novel BGCs (45 BGCs). In Lactobacillaceae, 40 of 73 species contained novel BGCs. *Lactiplantibacillus plantarum*, *Leuconostoc mesenteroides*, *Lactiplantibacillus paraplantarum*, *Latilactobacillus curvatus*, *Levilactobacillus brevis* and *Weissella paramesenteroides*_A contained more than 2 novel BGCs. Among these 40 Lactobacillaceae species, several, such as *L.* *plantarum*, *Leuconostoc pseudomesenteroides* and *Lacticaseibacillus rhamnosus*, were previously found to contain BGCs [45], suggesting unique characteristic associated with BGCs in intraspectic genotypes from food fermentations, which might be related with niche difference between food fermentations and other ecosystems.

### High divergent biosynthetic gene clusters in food fermentations compared with other ecosystems

**Fig. 4:**
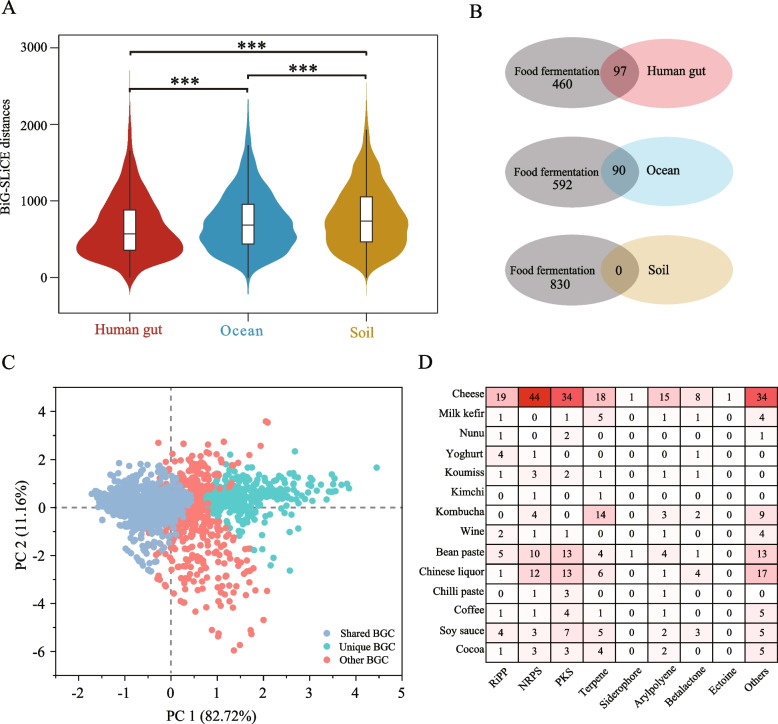
Divergence of biosynthetic gene clusters (BGCs) in food fermentations and three other ecosystems. **A** BiG-SLiCE distance of 2334 BGCs in food fermentations compared with BGCs from the human gut, ocean and soil ecosystems, respectively. The middle black lines in the violin plots are the average BiG-SLiCE distances. ***Represents *P* < 0.001. **B** The number of unique BGCs from species present in both food fermentations and other ecosystems and from species specifically present in food fermentations. **C** Principal component analysis (PCA) based on the BiG-SLiCE distance matrix of BGCs. Shared BGC represents the BGC present in food fermentations and all three ecosystems. Unique BGC represents the BGC present only in food fermentations. Other BGC represents the BGC present in food fermentations and one (or two) other ecosystems. **D** Distribution of unique BGCs from different BGC types in different food fermentation types. BGCs that are not included in the 8 dominant BGC types are combined and shown in Others

The human gut, ocean and soil ecosystems are considered important resources of BGCs for the development of bioactive compounds [13, 19, 20]. Considering the difference in niches, we systematically analysed the divergence of BGCs in food fermentations compared with these three ecosystems. We calculated the BiG-SLiCE distance of BGCs from food fermentations compared with those from the human gut, ocean and soil ecosystems (Fig. 4A), and a wide range distance was observed. The highest BiG-SLiCE distances of BGCs in food fermentations compared with those in the human gut, ocean and soil ecosystems were 2482, 2109 and 2312, respectively. This suggested that BGCs in food fermentations were distantly associated with those in three other ecosystems. Meanwhile, BiG-SLiCE distances were also significantly different between these three ecosystems (*P* < 0.001) using Wilcoxon rank-sum test. The BGCs in food fermentations showed a closer distance with those in human gut than the other two ecosystems. In addition, we analysed the taxonomic origins of 436 BGCs in food fermentations that had close distances (BiG-SLiCE distances < 300) with those from human gut. There were a total of 231 BGCs from 40 species in both food fermentations and human gut. Among these 231 BGCs, 164 BGCs (71.00%) were from 26 species that were previously reported as members of a healthy human gut microbiome (Supplementary Data 5).

Compared with BGCs from the human gut, the unique BGCs (BiG-SLiCE distance ≥ 900) included 43 RiPP, 100 NRPS, 118 PKS, 74 terpene, 5 siderophore, 47 arypolyene, 52 betalactone, 7 ectoine and 111 other BGCs in food fermentations. Among these BGCs, 460 (82.59%) unique BGCs were found in 145 species (Fig. 4B, Supplementary Fig. 10A) which were specifically found in food fermentations. A total of 97 (17.41%) unique BGCs were found in 25 species, e.g. *B.* *velezensis*, *L.* *lactis* and *Bacillus licheniformis*, which were present in both food fermentations and human gut (Supplementary Fig. 10A). This indicated that both the interspecific and intraspecies differentiation were associated with the divergence of BGCs, which might be resulted from the niche adaptation of MAGs. Compared with BGCs from the ocean, there were 682 unique BGCs in food fermentations. A total of 592 (86.80%) of these unique BGCs were found in 175 species specially in food fermentations, and 90 BGCs were found in 10 intraspecies genotypes within species present in both food fermentations and ocean (Supplementary Fig. 10B). Compared with BGCs from the soil, there were 830 unique BGCs in food fermentations. These BGCs were all found in 211 species specific to food fermentations (Supplementary Fig. 10C). These results indicated unique species played a vital role, and intraspecies differentiation played a secondary role in driving unique BGCs in food fermentations.

Compared with BGCs from all 3 other ecosystems, 419 BGCs (17.95%) were unique in food fermentations (Fig. 4C). These unique BGCs consisted of 8 dominant BGC types, including 40 RiPP, 84 NPKS, 83 PKS, 59 terpene, 2 siderophore, 32 arypolyene, 21 betalactone, 1 ectoine and 97 other BGCs. These 419 unique BGCs existed in 14 types of food fermentations (Fig. 4D). Cheese, bean paste and Chinese liquor fermentations all contained more than 50 unique BGCs, which accounted for 66.59% of the unique BGCs. There were 174 unique BGCs in cheese fermentation, and these unique BGCs were found in 56 species, of which 49 species were habitat-specific species in cheese fermentation. A total of 51 unique BGCs were found in bean paste fermentation, and these unique BGCs were found in 22 species, of which 13 species were habitat-specific species in bean paste fermentation. These results showed the unique BGCs widely distributed in different food fermentations, and the habitat-specific species mainly contributed to these unique BGCs in food fermentations.

### Prediction of secondary metabolites in food fermentations and their biological activities

**Fig. 5:**
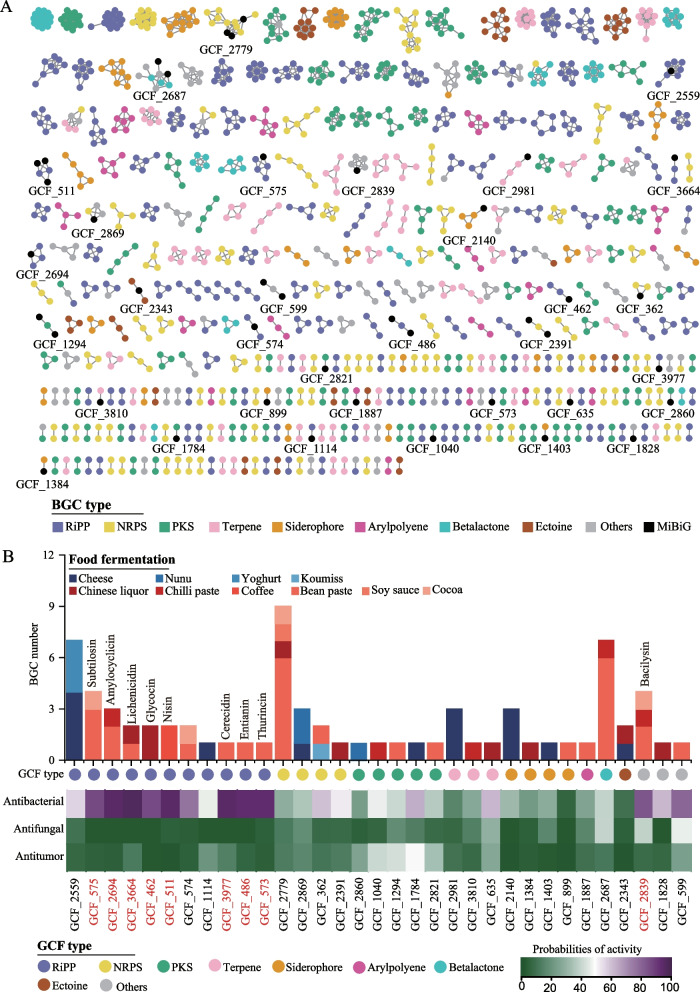
Prediction of secondary metabolites and their biological activities. **A** Gene cluster family (GCF) network of the 2334 identified biosynthetic gene clusters (BGCs). Each node represents one BGC. Only GCFs that contained more than one BGC are shown. BGCs that are not included in the 8 dominant BGC types are combined and shown in Others. Each cluster represents one GCF. The IDs of 33 GCFs that contain known BGCs from the MIBiG database are shown in the GCF network. **B** Prediction of biological activities for corresponding metabolite families of 33 known GCFs. The stacked columns represent the amounts and origins of BGCs in 33 GCFs. The heat map indicates the probabilities of different biological activities of 33 metabolite families. The metabolite families with high probabilities of antibacterial activity (> 80%) are shown on the stacked columns. The corresponding GCFs are highlighted in red

Exploring the composition of secondary metabolites in food fermentations would facilitate elucidating the health-beneficial effect of fermented foods. Minimum Information about a Biosynthetic Gene Cluster (MIBiG) database, containing BGCs with known secondary metabolites [46], helped reveal the secondary metabolites based on BGCs. The GCF approach, based on the similarity analysis of unstudied BGCs with reference BGCs in the MIBiG database [47], can be used to identify known secondary metabolites and their derivatives (named secondary metabolite families) produced by corresponding BGCs. We performed the GCF analysis to reveal the known secondary metabolites in food fermentations. Among 1415 GCFs in food fermentations, 33 GCFs contained known BGCs from the MIBiG database (Fig. 5A). These 33 GCFs consisted of 73 BGCs that belonged to 9 BGC types. The products annotated by these 73 BGCs were classified into 33 known secondary metabolite families (Supplementary Data 6).

To further analyse the biological activity of these known secondary metabolites in food fermentations, we performed a predicted analysis of the biological activity by a machine learning bioinformatics tool using BGC sequences [43]. The predicted probabilities of biological activities, including antibacterial, antifungal and antitumor activities, of 33 metabolite families were represented by the average value of their metabolite members. As shown in Fig. 5B, nine metabolite families, namely lichenicidin, amylocyclicin, cerecidin, nisin, entianin, thurincin, subtilosin, bacilysin and glycocin (produced by 20 BGCs), showed high probabilities of antibacterial activity (> 80%) (Supplementary Data 6). The lichenicidin family, showing the highest probability of antibacterial activity (98.00%), was found in both bean paste and Chinese liquor fermentations. Amylocyclicin was found in bean paste and chilli paste fermentations. Subtilosin was found in bean paste and cocoa fermentations. Bacilysin was found in bean paste, chilli paste and cocoa fermentations. The other 5 metabolite families were habitat-specific. Nisin was specific in coffee fermentation. Cerecidin, entianin and thurincin were specific in bean paste fermentation. Glycocin was specific in Chinese liquor fermentations, respectively. These results will contribute to revealing the health-beneficial potential of fermented foods.

The probability of biological activity was also observed for the products of unknown BGCs, ranging from 0.60 to 100% for antibacterial activity, from 2.84 to 52.26% for antifungal activity and from 0.11 to 49.30% for antitumor activity. The highest range of probability for antibacterial activity might be resulted from the most studies about the antibacterial activity of secondary metabolite among these different biological activities [43]. A total of 163 BGCs, containing 138 RiPP, 7 NRPS, 6 PKS, 6 terpene, 1 betalactone and 5 other BGCs, produced secondary metabolites with high probabilities of antibacterial activity (> 80%) (Supplementary Fig. 11A). These 163 BGCs were distributed across all 15 food fermentation types. Cheese fermentation contained the most BGCs (46 BGCs) producing secondary metabolites with high antibacterial activity probability, in which 31 BGCs were habitat-specific. Bean paste fermentation contained the second most BGCs (33 BGCs) producing secondary metabolites with high antibacterial activity probability, in which 21 BGCs were habitat-specific (Supplementary Fig. 11B). These results indicated a strong potential for antibacterial activity in these food fermentations.

## Discussion

Genome-resolved metagenomics of food fermentation samples allows the discovery of secondary metabolite BGCs and their taxonomic origins. Up to now, the biosynthetic potential of secondary metabolites was revealed in different representative ecosystems, such as human gut [19], oral [48], ocean [20], soil [13] and rumen [49] ecosystems. For food fermentation ecosystem, the biosynthetic potential of secondary metabolites was previously assessed by Walsh et al. [21] and Leech et al. [22], but these two studies only focused on the BGC-producing bacteriocins using BAGEL3 [50]. AntiSMASH uses a rule-based cluster detection approach and could identify 71 different types of secondary metabolite BGCs [39]. In this study, we used antiSMASH to provide a systematic and comprehensive analysis of the secondary metabolite BGCs in food fermentations. A total of 2334 BGCs were identified in these food fermentations. To the best of our knowledge, this study represents the largest investigation of BGCs in global food fermentations to date. Of note, the true biosynthetic potential of secondary metabolites might be underestimated in food fermentations because of the limitation of reference database. Collins et al. [51] noted that the profile of antibiotic-resistance genes in the intestinal microbiome of deep-sea fish was related with the novelty of antibiotic-resistance genes and the reference database used. AntiSMASH was rule based and might fail to detect unknown BGC types because of the lack of available library for unknown BGC types [14].

For the BGC distances between food fermentations and other ecosystems, we observed a closer distance between food fermentations and human gut. A lot of BGCs, which had close distances between these two ecosystems, were originated from the species reported as members of a healthy human gut microbiome. These species from food fermentations might be transferred to the human gut once the fermented foods were consumed. This study supported the links between food fermentations and human gut microbiome [52, 53].

There were discrepancies between MAGs reported in cheese and cocoa fermentations [21, 34] and MAGs recovered in this study. Besides the metagenomic dataset differences, the assembly and binning methods might also be main resaons for these discrepancies. IDBA-UD and MetaBAT2 were used for assembly and binning, respectively, in reported cheese fermentation samples [21], which were different with those in this study (MEGAHIT and metaSPAdes for assembly; Maxbin2, MetaBAT2 and CONCOCT for binning). Although the binning method in reported cocoa fermentation samples [34] was same with that in this study, it only used one assembler (MEGAHIT). Meanwhile, MAG quality cutoff would also be responsible for the MAGs discrepancies. The MAG quality cutoff was ≥ 50% completeness and ≤ 10% contamination in this study, which was in line with previous studies [54, 55]. In reported cocoa and cheese fermentation samples, the MAG quality cutoff were ≥ 50% completeness and < 10% contamination [34] and ≥ 80% completeness and ≤ 10% contamination [21], respectively. Here, we used the uniform quality cutoffs (≥ 50% completeness and ≤ 10% contamination) to filter these MAGs. Overall, the metagenomic dataset, assembly and binning methods and MAG quality cutoff value should all be considered for MAG recovery in different studies.

Identification of BCGs in food fermentations can not only provide novel insights into the potential human health benefits of fermented foods but also discover valuable secondary metabolites. The metabolites produced by BGCs have been one of most important sources of antibiotic drugs [56]. Culture-based techniques are usually difficult to discover novel secondary metabolites with novel chemical structures because many BGCs are silenced in laboratory conditions [17] or have variable expression patterns [57]. Identification of BCGs in food fermentations can serve to discover novel secondary metabolites using heterologous expression [58]. Meanwhile, prediction of biological activities of BGC-producing secondary metabolites could substantially aid in overcoming one of the primary barriers of secondary metabolite discovery: the prioritisation of BGCs for research.

Habitat-specific microbiota can be driven by environmental factors [59–61]. Raw material and processing method were considered as important factors driving the microbiota in food fermentations [22]. In this study, all the food fermentation samples were classified as milk- and plant-based fermentation groups. For the milk-based fermentation group, raw material is mainly milk, but the processing method is different. For instance, cheese fermentation consists of the removal of whey, which is not done in yoghurt fermentation [1]. Therefore, the processing method can be a key factor driving the differentiation of BGC-contained microbial taxon in the milk-based fermentation group. For the plant-based fermentation group, raw material and processing method are both different. For instance, bean paste is produced by semi-solid-state fermentation with soybean as raw material [62], coffee is produced by solid-state fermentation with coffee bean as raw material [63] and Chinese liquor is produced by solid-state fermentation with grains, such as sorghum, as raw material [64]. Therefore, both raw material and processing method can be key factors driving the differentiation of BGC-contained microbial taxon in the plant-based fermentation group.

Biological activities for secondary metabolites based on their BGC sequences were predicted by a machine-learning bioinformatics tool [43]. In this study, nine metabolite families were predicted to have high probability of antibacterial activity (> 80%), and the predicted antibacterial activity was consistent with a previous study (subtilosin [65], amylocyclicin [66], lichenicidin [67], glycocin [68], nisin [69], cerecidin [70], entianin [71], bacilysin [72] and thurincin [73]). Certain secondary metabolites, such as lichenysin [74], difficidin [75] and bacillibactin [76], were previously reported to have antibacterial activities. However, their probabilities of antibacterial activity were only predicted to be 60.00%, 36.21% and 27.57%, respectively, in this study. Therefore, we should isolate strains containing these BGCs, or heterogeneously express these BGCs to obtain metabolites, to confirm or characterise the activities of these BGC-producing secondary metabolites in vitro. Meanwhile, the present prediction method can only predict three types of biological activity. Other biological activities, such as antioxidant, antiviral and antiprotozoal activities, should also be analysed by in vitro experiments in the future. In addition to the known secondary metabolites, there were many secondary metabolites produced by unknown BGCs. For instance, 871 unknown BGCs were identified in cheese fermentations, but only 15 BGC-producing secondary metabolites belonged to known metabolite families. It would be beneficial to elucidate the potential health benefits of fermented foods by investigating the chemical structures and biological activities of these unknown secondary metabolites produced by unknown BGCs. Moreover, the concentrations of these metabolites in fermented foods should also be determined, which would facilitate formulating proper intake of these fermented foods. Meanwhile, establishing the metabolic pathways of the secondary metabolites would serve to regulate these compounds in food fermentations, consequently accelerating the development of a variety of new healthy fermented foods.

In this study, we relied on 653 MAGs recovered from metagenomic sequencing data to predict BGCs. The MAG approach is proved to be an efficient tool to explore secondary biosynthetic potential in different food fermentations. However, compared with the whole genome analysis for BGCs, the MAG approach could generate more incomplete BGCs and was unfriendly to low-abundance species [77]. This issue would be resolved to some extent by increasing the sequencing depth and improving the sequencing method, such as using a third-generation sequencing method [77]. In addition, this study analysed metagenomic sequencing data from 367 fermented samples belonging to 15 food fermentation types. Although the metagenomic sequencing data we analysed here spanned fermentations of general fermented foods, there are currently more than 200 types of fermented foods with different origins and processing ways worldwide [1]. As a result, we will further collect metagenomic sequencing data from more food fermentation types to reveal BGCs in global food fermentations.

## Conclusion

To conclude, this study revealed that food fermentation was an untapped reservoir of secondary metabolite BGCs, including a lot of BGCs corresponding secondary metabolites with high probabilities of antibacterial activity. Secondary metabolite BGCs widespreadly and habitat-specifically distributed in different food fermentation types driven by both habitat-specific species and intraspecies genotypes. This study would serve to elucidate the health-beneficial potential of fermented foods and develop novel bioactive compounds from food fermentations.

## Supplementary Information

**Additional file 1:** **Supplementary Data 1.** Description of the metagenomic sequencing data of 367 food fermentation samples.

**Additional file 2:** **Supplementary Data 2.** The taxonomic annotation of 653 metagenome-assembled genomes (MAGs).

**Additional file 3:** **Supplementary Data 3.** The statistical analysis for the associations between phyla/clusters/BGC types and food fermentation types/groups.

**Additional file 4:** **Supplementary Data 4.** Two thousand three hundred thirty-four biosynthetic gene clusters (BGCs) and their distributions in gene cluster families (GCFs).

**Additional file 5:** **Supplementary Data 5.** The members of a healthy human gut microbiome in the 40 shared species between food fermentations and human gut.

**Additional file 6:** **Supplementary Data 6.** Seventy-three known biosynthetic gene clusters (BGCs) and predicted biological activities of their products.

**Additional file 7:** **Supplementary Fig. 1.** Sequencing depth of each food fermentation type. **Supplementary Fig. 2.** CheckM quality assessment. **Supplementary Fig. 3.** The BGC numbers of each metagenome-assembled genome (MAG) in different food fermentations. **Supplementary Fig. 4.** Ratio of all biosynthetic gene clusters (BGCs) and novel BGCs from unknown metagenome-assembled genomes (MAGs) compared with all MAGs. **Supplementary Fig. 5.** Biosynthetic gene cluster (BGC) number of each metagenome-assembled genome (MAG) in different families. **Supplementary Fig. 6.** Venn diagram showing the distribution of biosynthetic gene clusters (BGCs) across habitat-specific BGCs and BGCs from habitat-specific species. **Supplementary Fig. 7.** Distribution of 1,655 habitat-specific biosynthetic gene clusters (BGCs) from habitat-specific and multi-habitat species. **Supplementary Fig. 8.** Distribution of novel biosynthetic gene clusters (BGCs) in different food fermentations. **Supplementary Fig. 9.** Nested bubble diagram showing the ratio of novel biosynthetic gene clusters (BGCs) to all BGCs. **Supplementary Fig. 10.** Distribution of unique biosynthetic gene clusters (BGCs) in food fermentations compared with human gut (A), ocean (B) and soil (C) ecosystems. **Supplementary Fig. 11.** Prediction of biological activities of secondary metabolites produced by unknown biosynthetic gene clusters (BGCs).

## Data Availability

The metagenomic data used in this study were downloaded from NCBI, and a summary of their accessions was provided in Supplementary Data 1. Three-hundred twenty-eight publicly available MAGs from cheese fermentation were downloaded from customised Google Drive (https://drive.google.com/file/d/1TCLYBX7kkxNUWn4jr4YGXNL_qV97lc70/view). Twenty-nine publicly available MAGs from cocoa fermentation were downloaded from a customised GitHub repository (https://github.com/Otavio20/Cocoa_MAGs). The MIBiG and BiG-FAM databases can be accessed at https://mibig.secondarymetabolites.org/ and https://bigfam.bioinformatics.nl/, respectively. The data produced in this study, including 653 MAGs and 2334 BGCs, had been deposited and were available at the GitHub repository (https://github.com/durubing-jn/food-fermentation-mategenome). The codes for metagenomic sequencing data assembly and binning were available at the GitHub repository (https://github.com/durubing-jn/food-fermentation-mategenome).
