# Supplementary material for: Metagenomics reveals the habitat specificity of biosynthetic potential of secondary metabolites in global food fermentations
Source: Microbiome. 2023 May 20;11:115. doi: 10.1186/s40168-023-01536-8 (PMC10199516; doi:10.1186/s40168-023-01536-8)
Supplement: Supplementary file 8 — Additional file 7: Supplementary Fig. 1. Sequencing depth of each food fermentation type. Supplementary Fig. 2. CheckM quality assessment. Supplementary Fig. 3. The BGC numbers of each metagenome-assembled genome (MAG) in different food fermentations. Supplementary Fig. 4. Ratio of all biosynthetic gene clusters (BGCs) and novel BGCs from unknown metagenome-assembled genomes (MAGs) compared with all MAGs. Supplementary Fig. 5. Biosynthetic gene cluster (BGC) number of each metagenome-assembled genome (MAG) in different families. Supplementary Fig. 6. Venn diagram showing the distribution of biosynthetic gene clusters (BGCs) across habitat-specific BGCs and BGCs from habitat-specific species. Supplementary Fig. 7. Distribution of 1,655 habitat-specific biosynthetic gene clusters (BGCs) from habitat-specific and multi-habitat species. Supplementary Fig. 8. Distribution of novel biosynthetic gene clusters (BGCs) in different food fermentations. Supplementary Fig. 9. Nested bubble diagram showing the ratio of novel biosynthetic gene clusters (BGCs) to all BGCs. Supplementary Fig. 10. Distribution of unique biosynthetic gene clusters (BGCs) in food fermentations compared with human gut (A), ocean (B) and soil (C) ecosystems. Supplementary Fig. 11. Prediction of biological activities of secondary metabolites produced by unknown biosynthetic gene clusters (BGCs). [file 40168_2023_1536_MOESM7_ESM.docx]

# Title: Metagenomics reveals the habitat specificity of biosynthetic potential of secondary metabolites in global food fermentations

Rubing Du ^a^, Wu Xiong ^b^, Lei Xu ^a^, Yan Xu ^a^, Qun Wu ^a*^

^a^ Lab of Brewing Microbiology and Applied Enzymology, The Key Laboratory of Industrial Biotechnology, Ministry of Education; State Key Laboratory of Food Science and Technology; School of Biotechnology, Jiangnan University, Wuxi, 214122, Jiangsu, People’s Republic of China.

^b^ Jiangsu Provincial Key Lab of Solid Organic Waste Utilization, Jiangsu Collaborative Innovation Center of Solid Organic Wastes, Educational Ministry Engineering Center of Resource-saving fertilizers, Laboratory of Bio-interactions and Crop Health, Nanjing Agricultural University, Nanjing, 210095, Jiangsu, People’s Republic of China.

* Corresponding author:

# E-mail: wuq@jiangnan.edu.cn (Q. Wu)

# Supplementary Fig. 1–11


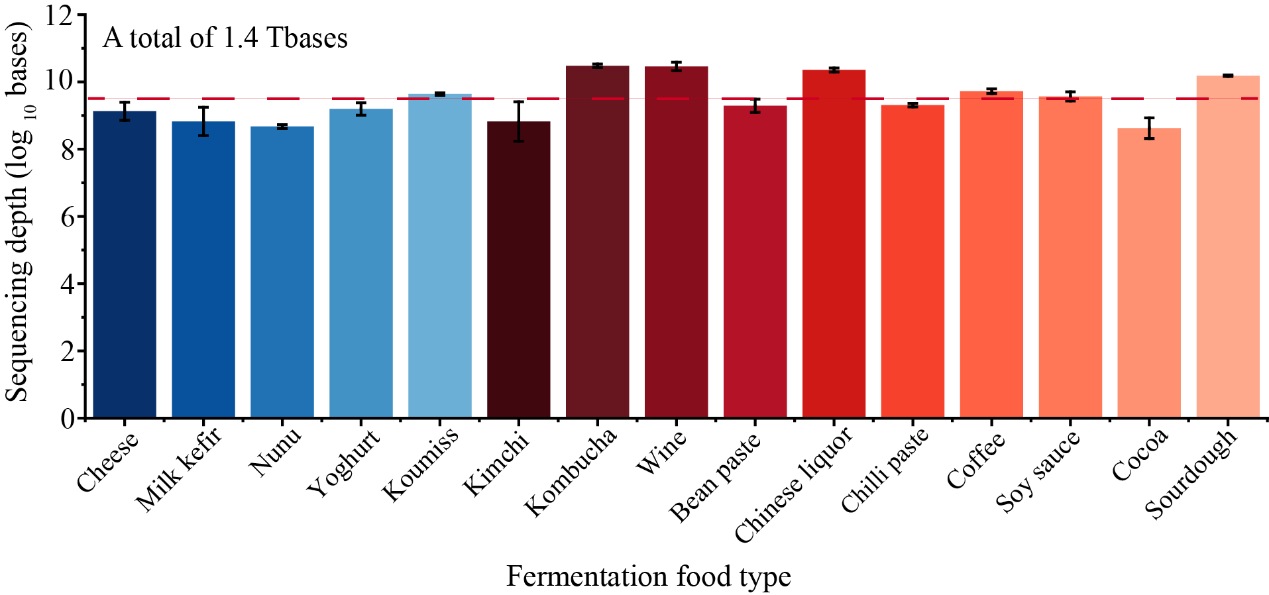


**Supplementary Fig. 1** **Sequencing depth of each food fermentation type.** The red line represents the average sequencing depth.


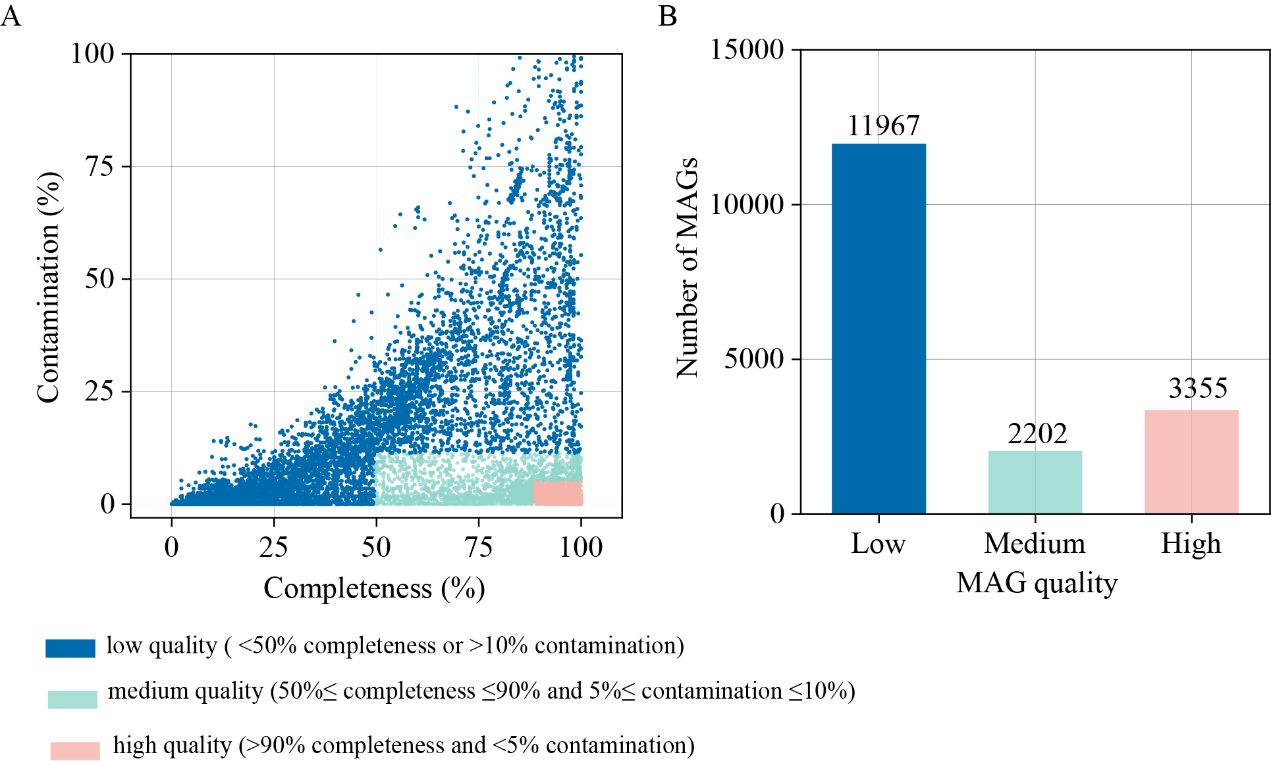


**Supplementary Fig. 2 CheckM quality assessment.** (A) The quality assessment of the 17,524 metagenome-assembled genomes (MAGs). (B) The number of MAGs recovered according to the levels of genome completeness and contamination.


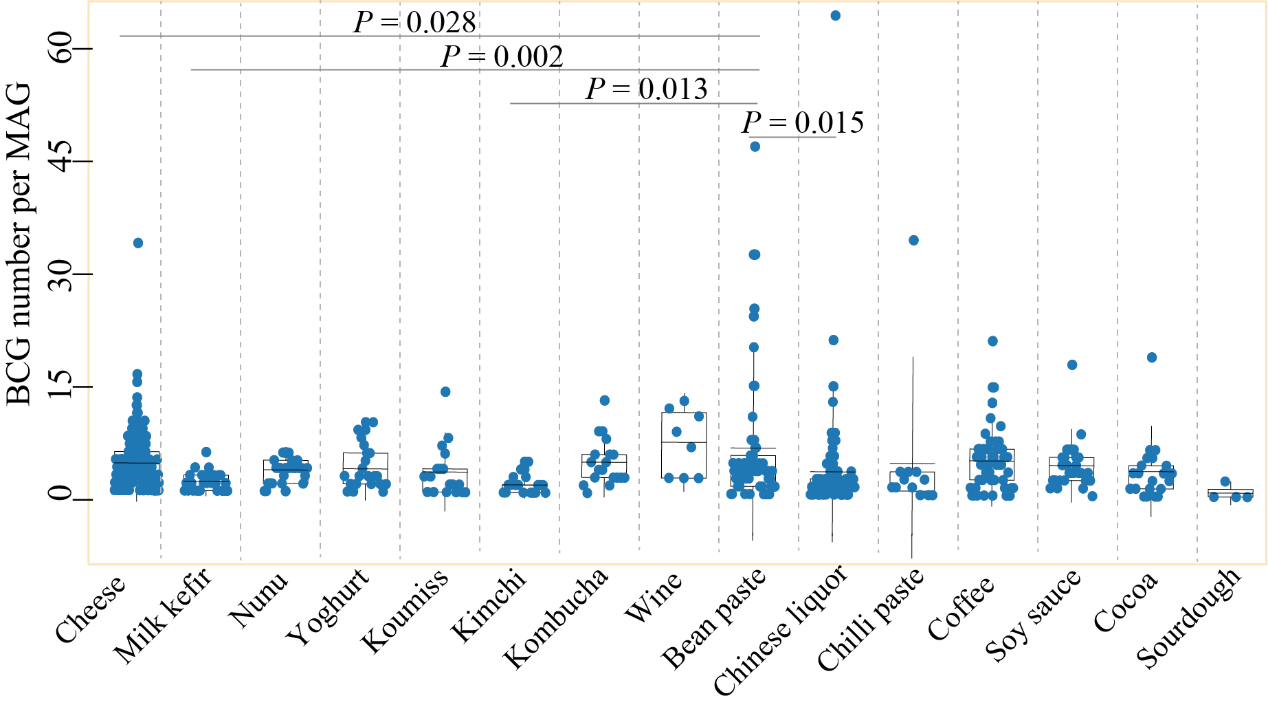


**Supplementary Fig. 3 The BGC numbers of each metagenome-assembled genome (MAG) in different food fermentations.** The black lines represent the mean BGC numbers per MAG.


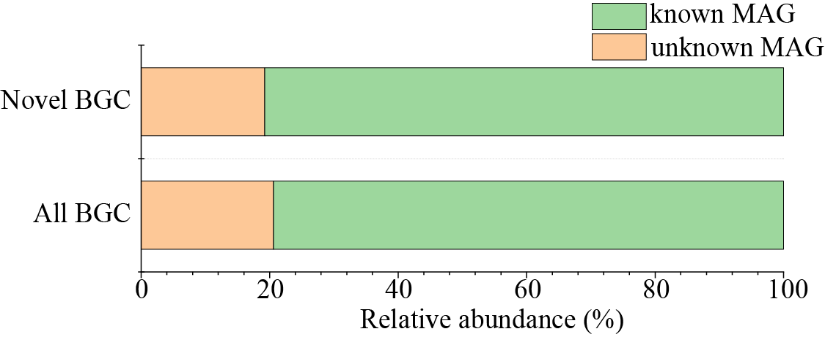


**Supplementary Fig. 4 Ratio of all biosynthetic gene clusters (BGCs) and novel BGCs from unknown metagenome-assembled genomes (MAGs) compared with all MAGs.**


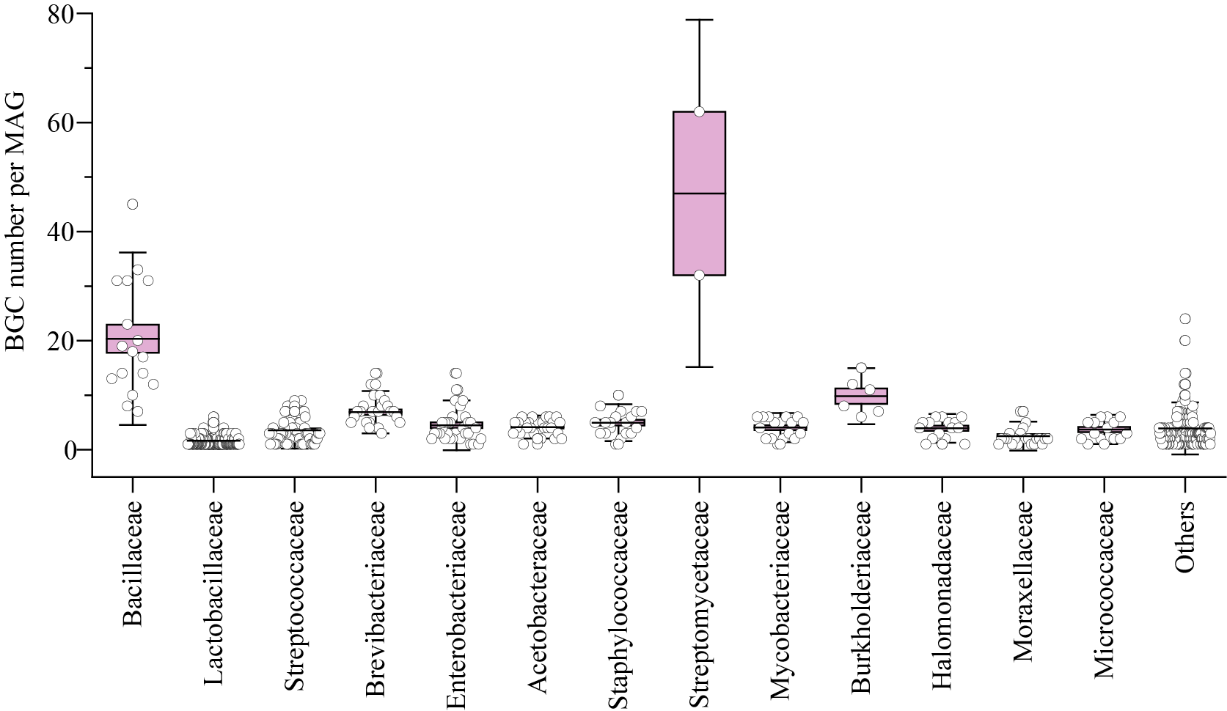


**Supplementary Fig. 5 Biosynthetic gene cluster (BGC) number of each metagenome-assembled genome (MAG) in different families.** In the boxplots, the black lines represent the average BGC numbers per MAG in different families. Species that are not included in the 13 BGC-rich families are combined and shown in Others.


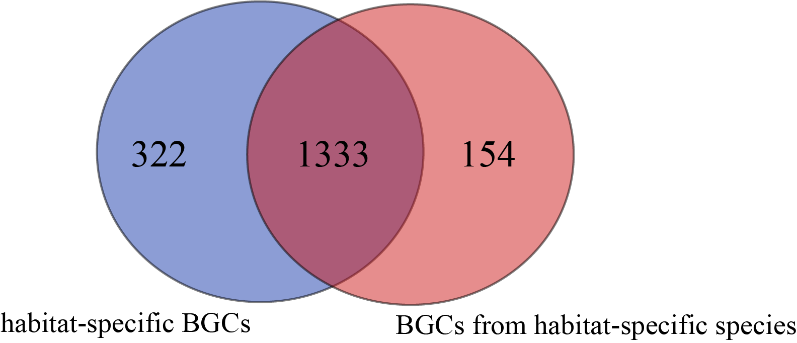


**Supplementary Fig. 6 Venn diagram showing the distribution of biosynthetic gene clusters (BGCs) across habitat-specific BGCs and BGCs from habitat-specific species.**


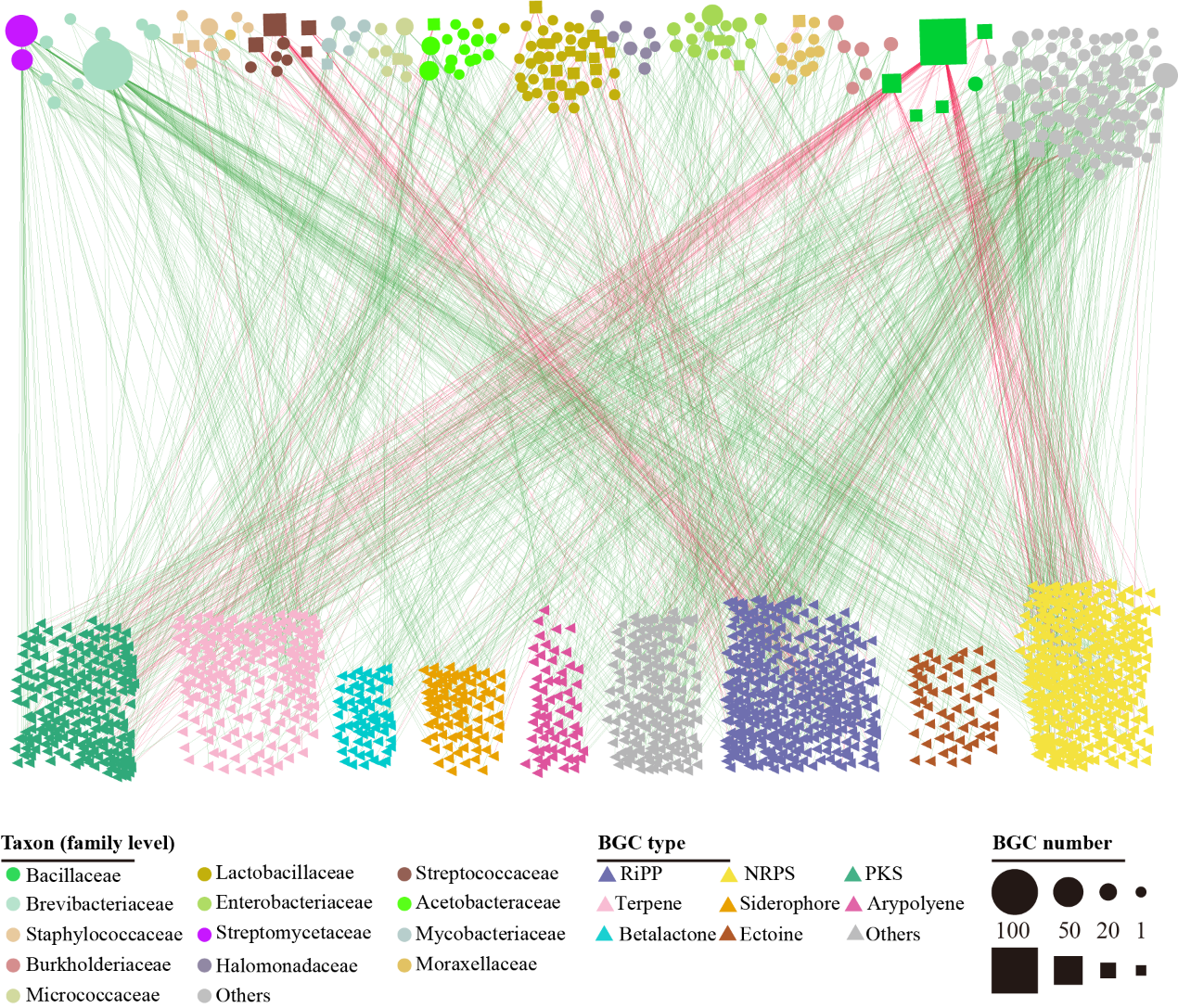


**Supplementary Fig. 7 Distribution of 1,655** **habitat-specific biosynthetic gene clusters (BGCs) from** **habitat-specific and multi-habitat species.** The triangles represent BGCs. The circles and squares represent habitat-specific and multi-habitat species, respectively. The size of the circles and squares represents the BGC number in the individual species. BGCs that are not included in the 8 dominant BGC types are combined and shown in Others. Species that are not included in the 13 BGC-rich families are combined and shown in Others. The green lines represent that the taxonomic origins of BGCs are habitat-specific species. The red lines represent that the taxonomic origins of BGCs are multi-habitat species.


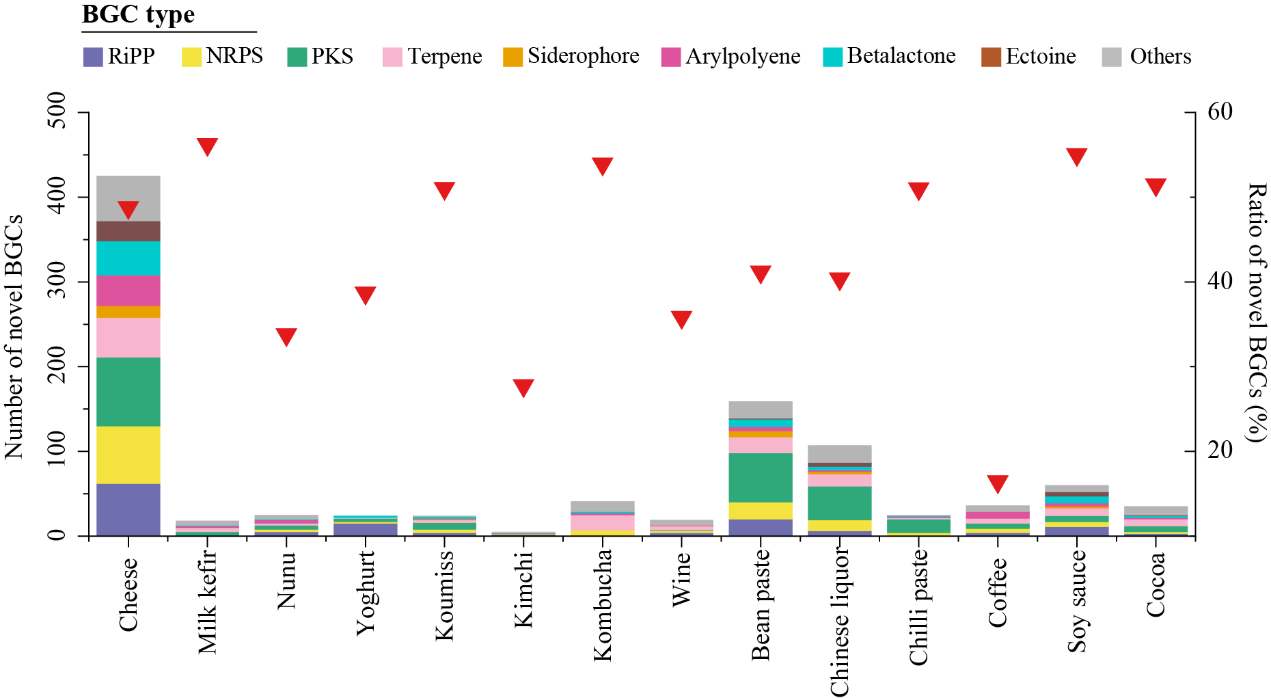


**Supplementary Fig. 8** **Distribution of novel biosynthetic gene clusters (BGCs) in different food fermentations.** The stacked columns represent the abundances of different BGC types in different food fermentation types. The triangles represent the ratios of novel BGC number to total BGC number in different food fermentation types. BGCs that are not included in the 8 dominant BGC types are combined and shown in Others.


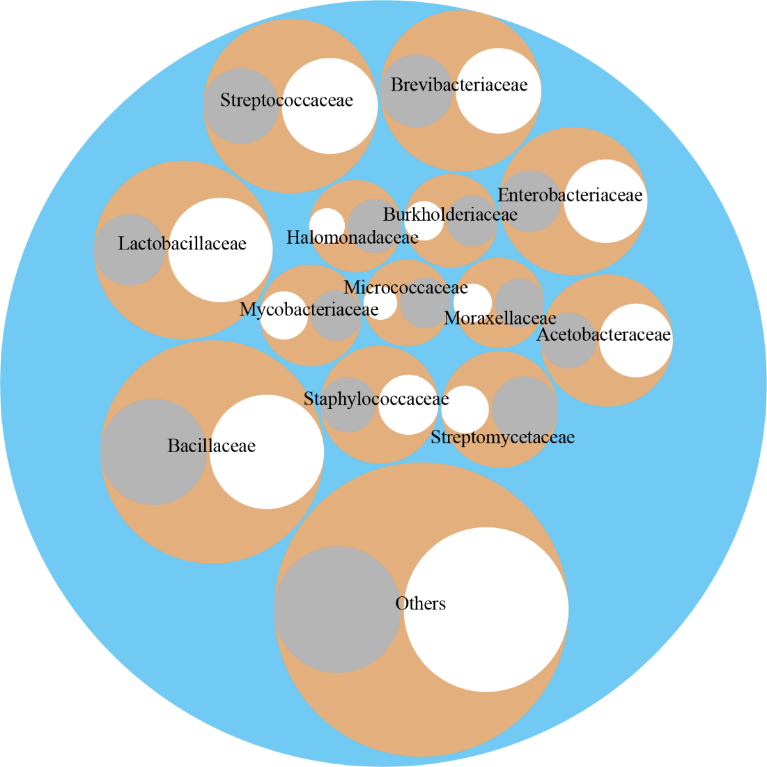


**Supplementary Fig. 9 Nested bubble diagram showing the ratio of novel biosynthetic gene clusters (BGCs) to all BGCs.** The largest bubble in blue represents all BGCs. The inner brown bubbles represent BGCs in different families. The innermost bubbles in grey and white represent novel and known BGCs, respectively. Species that are not included in the 13 BGC-rich families are combined and shown in Others. The size of the bubble is proportional to the BGC number.


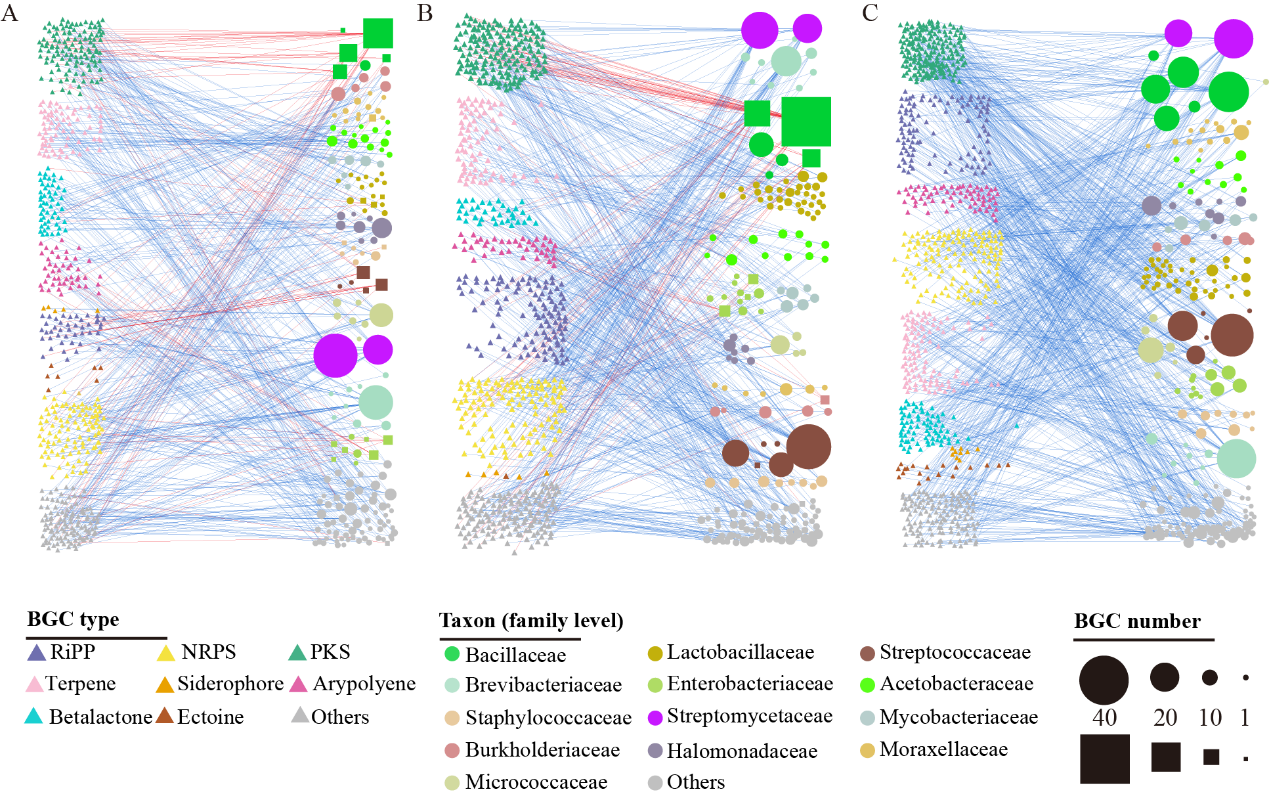


**Supplementary Fig. 10 Distribution of unique biosynthetic gene clusters (BGCs) in food fermentations compared with human gut (A), ocean (B) and soil (C) ecosystems.** The triangles represent BGCs. The circles represent unique BGC-containing species present specifically in food fermentations, and the squares represent unique BGC-containing species present in both food fermentations and other ecosystems. The size of the circles and squares represents the BGC number in the individual species. BGCs that are not included in the 8 dominant BGC types are combined and shown in Others. Species that are not included in the 13 BGC-rich families are combined and shown in Others. The blue lines represent that the taxonomic origins of BGCs are species-specific in food fermentations. The red lines represent that the taxonomic origins of BGCs are species present in both food fermentations and other ecosystems.


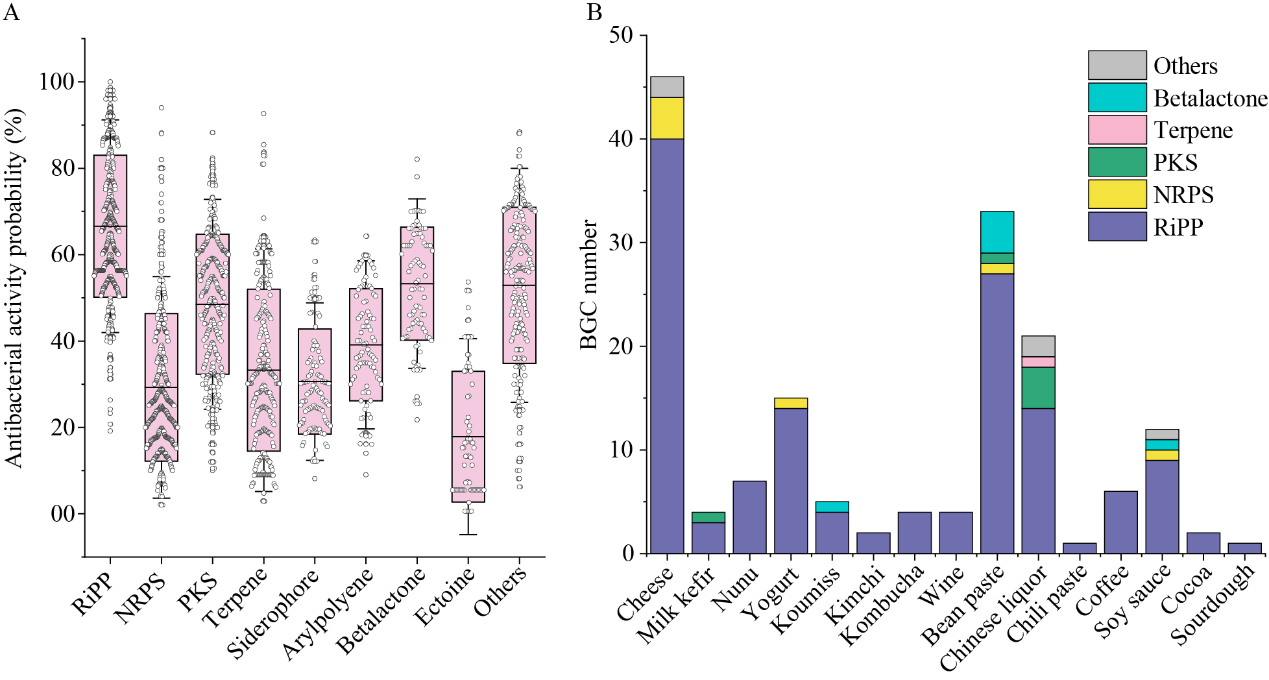


**Supplementary Fig. 11 Prediction of biological activities of secondary metabolites produced by unknown biosynthetic gene clusters (BGCs).** (A) Probability of antibacterial activity of metabolites produced by unknown BGCs. The black lines in the boxplots are the average probabilities of antibacterial activity of the metabolites of different BGC types. BGCs that are not included in the 8 dominant BGC types are combined and shown in Others. (B) Stacked columns showing the distributions of unknown BGCs that produced metabolites with high probabilities of antibacterial activity (>80%) in different food fermentation types.
